# Use of food distribution resources among military families with young children since the COVID-19 pandemic

**DOI:** 10.1017/S1368980023001738

**Published:** 2023-10

**Authors:** Catherine W O’Neal, Mallory Lucier-Greer, Crystal Lewis, Meredith Farnsworth

**Affiliations:** 1 Department of Human Development and Family Science, 202 Family Science Center II, University of Georgia, Athens, GA 30602, USA; 2 Department of Human Development and Family Science, 203 Spidle Hall, Auburn University, Auburn, AL 36849, USA; 3 Independent, San Diego, CA, USA; 4 National Military Family Association, 2800 Eisenhower Avenue, Suite 250, Alexandria, VA 22314, USA

**Keywords:** Food distribution resources, Community sample, Military, Family

## Abstract

**Objective:**

The present study examined military families’ use of food distribution resources and military (e.g. rank) and non-military (e.g. race/ethnicity) characteristics associated with using food distribution resources.

**Design:**

Secondary data analyses from a cross-sectional survey in the first 6 months of 2021.

**Setting:**

A national sample of eligible families completed an online survey.

**Participants:**

8326 enlisted military families with an active duty service member in the United States Army or Air Force who applied for supplemental childcare funding distributed by National Military Family Association.

**Results:**

13·2 % of the families reported utilising a food distribution resource in the past 12 months. Those with lower financial well-being were more likely to utilise such resources. Older (OR = 1·04, 95 % CI = 1·02, 1·05, *P* < 0·001), single-earner (OR = 0·73, 95 % CI = 0·61, 0·89, *P* = 0·001) families with a lower rank (OR = 0·69, 95 % CI = 0·64, 0·75, *P* < 0·001) and Army affiliation (compared with Air Force) (OR = 2·31, 95 % CI = 2·01, 2·67, *P* < 0·001) were more likely to utilise food distribution resources. Members of certain racial/ethnic minority groups were more likely to utilise food distribution resources than White respondents (OR from 1·47 for multi-racial to 1·69 for Asians), as were families with more dependent children (OR = 1·35, 95 % CI = 1·25, 1·47, *P* < 0·001).

**Conclusions:**

These results identify the extent of food distribution resource utilisation in military families with young children approximately 1 year into the COVID-19 pandemic. The results also identify characteristics associated with their use of food distribution resources. Findings are discussed with an emphasis on prevention and intervention implications for military families.

Food insecurity is a public health concern with detrimental effects on both physical and mental health, and it has been exacerbated by the COVID-19 pandemic^(1)^. The transitions military families face (e.g. prolonged separations and frequent relocations) may further increase their risk of experiencing food insecurity. Previous research has assessed the prevalence of food insecurity among military families^(1)^. However, it is necessary to reconsider food insecurity for military families since the COVID-19 pandemic, particularly families with young children, as they are often at a greater risk for food insecurity and subsequent physical and mental health challenges^(2)^. Relatedly, there is a need to better understand at-risk families’ use of food distribution resources. The current study aims to extend previous research by (1) addressing the prevalence of resource utilisation intended to offset food insecurity, particularly the use of charitable food distribution resources (hereafter referred to as food distribution resources), among military families with young children in the spring of 2021 (since the COVID-19 pandemic), (2) examining the extent of the association between their use of food distribution resources and their financial well-being and (3) identifying military and non-military characteristics associated with families’ use of food distribution resources (e.g. rank and race/ethnicity).

Food insecurity describes households that lack adequate resources to access the appropriate variety, quality and/or amount of food to maintain a healthy lifestyle^(3)^. In May 2020, an estimated 20·1 % of U.S. households with children were food insecure^(4)^. This estimate was approximately three percentage points higher than estimates from 2016 to 2017 (before the pandemic). Food insecurity represents a public health risk as it can have detrimental effects on physical health (e.g. being more likely to experience issues such as oral health problems, diabetes, and hypertension^(5)^) and mental health (e.g. being more likely to experience depression, anxiety and sleep disorders^(6,7)^) across the lifespan.

## Military families as a vulnerable group

Even before the economic hardship associated with the COVID-19 pandemic, military families were identified as a vulnerable group at elevated risk of experiencing food insecurity^(8)^. In 2015, from a sample of 248 military families with an active-duty service member and young children, approximately one in seven (∼14·3 %) military families reported experiencing food insecurity^(1)^. In a 2022 study of Army families during the pandemic, approximately one in three (33 %) military families experienced marginal food insecurity^(9)^. These studies demonstrate that although some military families were experiencing food insecurity prior to the COVID-19 pandemic, the pandemic was linked to an increase in the number of military families experiencing food insecurity, likely due to financial strains experienced during the pandemic.

In addition to food insecurity as a nationwide public health crisis, for military families, food insecurity is a national defense concern. Research indicates that service members’ intentions to remain in the military are related to their experiences of food insecurity and mental health^(10)^. More specifically, with a sample of 5677 Army soldiers, those experiencing food insecurity were found to have lower mental health. In turn, they were more likely to report intentions to leave the military after the current contract^(10)^. The prevalence of food insecurity for military families along with the implications of food insecurity for families and, more broadly, national defense (in terms of service member retention and mental health), prompted food distribution resources geared towards military families specifically. However, the extent to which these resources are utilised by military families is unclear. Furthermore, little is known about differences in military families’ use of such resources considering military and non-military characteristics (e.g. rank, single-earner families).

## Factors related to food insecurity and related resource utilisation

Knowing more about who utilises food distribution resources can position policymakers and helping organisations to develop and implement resources that effectively address food insecurity for military families. Research, to date, has largely not examined factors related to the use of resources intended to address food insecurity, particularly for military families. However, existing research has identified military and non-military characteristics that may increase military families’ risk of food insecurity, which may shed some light on who is utilising resources. For instance, a service member’s *rank* is analogous to paygrade or income and is largely determined by educational attainment and years of service. Enlisted service members (who must have a high school diploma or pass the General Education Development test before joining the military) are more likely to experience food insecurity than officers (who complete a bachelor’s degree before being commissioned in the military)^(9)^. Other military characteristics have received less empirical attention in connection to food insecurity and warrant more research. For instance, *transitions* related to deployment or other assignments that take the service member away from the family system for a period of time may also impact food insecurity and related resource utilisation because they often alter the service member’s pay and family norms (e.g. spending behavior). *Housing location* (e.g. living on or off the installation) is another factor to consider because it may reflect greater access to resources^(11)^. There can be differences by branch of service as well^(11)^.

The prevalence of food insecurity has also been shown to vary systematically by individual and family characteristics that are not specific to the military context. For example, *younger* individuals and *single-parent households* are more likely to report being food insecure^(1,6,9,12)^. Additionally, in civilian and military samples, *racial/ethnic minorities*, particularly African Americans, and those with lower *educational attainment* (e.g. high school, GED, or lower) are at elevated risk for experiencing food insecurity^(6,9,13)^. Households with *more dependent children* also generally report greater food insecurity regardless of military status^(6,9)^.

Characteristics related to household income, such as *single-earner families*, are key factors related to food insecurity^(14)^. Even with the financial stability of the service member’s income, military spouses are more likely to be unemployed or underemployed compared with civilians^(15)^; thus, military families tend to spend at least some periods of time as single-earner families. Previous studies of military families have demonstrated a link between having fewer full-time income earners in the home and food insecurity^(1,9)^. Further, military partners are primarily women^(16)^, and employment among women has been disproportionately affected by the COVID-19 pandemic^(17)^.

## Current study

The current study extends previous research focused on military families’ food insecurity to provide insight into the prevalence of food distribution resource use among military families in the United States (U.S.) with young children since the COVID-19 pandemic with data from a large, national sample. Furthermore, the study assesses how food distribution resource use varies by families’ financial well-being and identifies military (i.e. current deployment status, living on a military installation, rank and service branch) and non-military characteristics (i.e. age, race/ethnicity, single-parent household, number of children in the household and dual-earner family) associated with their use of food distribution resources. Enhanced knowledge of military families’ use of food distribution resources can shed light on which families locate and utilise such resources, with implications for better bridging the gap between families who need and utilise resources.

## Methods

### Participants and procedures

The current study used data from 8326 U.S. military families who applied for $1500 to supplement their childcare costs in the spring of 2021. The supplemental funds were distributed by the National Military Family Association as part of the military’s efforts to alleviate challenges military families faced related to childcare during the COVID-19 pandemic^(18)^. To apply for the supplemental funds, service members or their partners completed the electronic survey. The application was open to Air Force and Army families with a rank between E-1 and E-6 serving on active-duty orders. This includes active-duty (i.e. a full-time) service members and activated Guard and Reserve members (i.e. temporarily serving as active duty). With the application, they submitted the service members’ leave and earning statement (i.e. paycheck) as proof of their military status. For married service members, civilian spouses had to be working full-time, attending school (full- or part-time) or pursuing a professional license or certificate (minimum of 15 h per week commitment). Proof of work/education status was not required. Funds were provided to the families to use for any manner of childcare expense (e.g. full-time, part-time and/or babysitting care provided by a licensed centre or individual), though receipts were required as proof of childcare expenses. The supplement was provided to all families who met these eligibility criteria until the available funds were exhausted. The supplemental childcare funding was promoted via emails to National Military Family Association program participants and newsletters, social media and partner organisations. The electronic survey was created by researchers within National Military Family Association, and deidentified data from the survey were utilised for this secondary data analysis.

### Measures

#### Charitable food distribution utilisation

Respondents indicated if anyone in their household ‘had to visit a charitable distribution site to make ends meet in the past 12 months’ (yes = 1; no = 0).

#### Financial well-being

Respondents completed the ten-item Consumer Financial Protection Bureau’s Financial Well-being Scale^(19)^, which assessed four elements of financial well-being, including control over day-to-day finances, capacity to absorb a financial shock, being on track with financial goals and financial freedom to make choices allowing life enjoyment. Sample items include ‘I am just getting by financially’ and ‘I am behind with my finances’. Items were scored on a five-point scale with responses for six questions ranging from ‘not at all’ to ‘completely’ and the remaining four questions ranging from ‘never’ to ‘always’. Following the scale scoring instructions, some items were reverse coded, and the sum scores were calculated. Then, sum scores were converted to total response values normed based on population responses, with values ranging from 14 to 86. Higher values indicate greater financial well-being. The scale demonstrated high reliability (*α* = 0·87).

#### Military characteristics

Four military characteristics were examined, including the service members’ *rank* (i.e. paygrade) (ranging from 1 = E-1 to 6 = E-6), the service member parent experiencing a *military assignment* away from their home duty station, such as being currently deployed or on temporary duty assignment (TDY) (1 = yes; 0 = no), *living on a military installation* (1 = yes; 0 = no) and *service branch* (1 = Army; 0 = Air Force).

#### Non-military characteristics

Five non-military individual and family characteristics were examined. Respondents indicated their *age* as a continuous variable. *Race/ethnicity* was dummy coded as a series of binary variables (yes = 1; no = 0) indicating ‘Asian,’ ‘Black,’ ‘Hispanic/Latino/a/e,’ ‘White,’ ‘Other’ and ‘Multi-racial.’ White, the race/ethnicity variable with the highest frequency, was utilised as the reference group. Respondents indicated if they were a *single parent* (1 = yes, 0 = no) and the *number of dependent children* in their household (ranging from 1 = 1 child to 5 = 5 or more dependent children). *Dual-earner family* (1 = yes; 0 = no) was coded as respondents indicating that they and their partner were employed full- or part-time.

#### Analytic strategy

Descriptive statistics were examined to identify the prevalence of utilisation of charitable food distribution resources in this national sample of military families with young children. To establish the extent to which visiting a food distribution site in the last 12 months was associated with financial well-being, a one-way ANOVA was conducted to explore the mean score differences in financial well-being between military families who used and did not use charitable food distribution resources. A binomial logistic regression was utilised to identify military and non-military characteristics related to using food distribution resources. More specifically, the four military characteristics and five non-military characteristics were simultaneously entered as independent variables, with charitable food resource utilisation as the binary dependent variable. Although the model produces beta estimates as logit values, odds ratios (OR) (i.e. exponentiated logit values) are the more interpretable, and commonly reported, results^(20)^. OR indicate the likelihood of using food distribution resources based on each ‘predictor’ variable (e.g. rank). A statistically significant OR greater than 1 indicates an increased likelihood of resource use, while an OR less than 1 indicates a lower likelihood of resource use. Including the military and non-military characteristics simultaneously in the regression analysis enables the estimation of unique effects of each predictor variable after controlling for (i.e. adjusting for) all other variables in the equation. Many common measures of overall model fit are not available for logistic regression models. However, the Hosmer and Lemeshow test for model fit is available. Non-significant values indicate that the data are a sufficient fit to the tested model (more specifically, this test evaluates if the predicted probabilities are different from the observed probabilities)^(20)^.

## Results

Descriptive statistics indicated that 13·2 % of the military families with young children (*n* 1101; slightly over 1 in 8 families) reported utilising a charitable food resource in the past 12 months. Over half of the survey respondents were women (59·7 %), with a mean age of 29·91 years (sd = 4·89). The most commonly reported races/ethnicities included: White (40·5 %), Black (24·5 %) and Hispanic (14·0 %). Most respondents were married (74·4 %). All participating families had at least one dependent child; most families averaged 1 or 2 dependent children (82·4 %). Almost two-thirds of the families were two-income families (63·0 %). Most families included an active-duty service member (85·1 %), followed by activated members of the National Guard (12·0 %) or Reserve (3·0 %). Two-thirds of the families were affiliated with the Air Force (69·4 %), and the most common ranks were E-5 (35·8 %) and E-6 (37·5 %). Almost three-fourths (72·7 %) of the families lived off the military installation. One in ten (11·5 %) families was experiencing a deployment or an extended temporary duty assignment at the time of data collection. See Table 1 for the full sample’s characteristics and the sample stratified by charitable food resource use in the past 12 months.

**Table 1:** Sample characteristics (*n* 8326)

Characteristics	Charitable food resource utilisation					
	Yes (*n* 1101; 13·2 %)		No (*n* 7225; 86·8 %)		Total	
	%	*n*	%	*n*	%	*n*
Gender						
Male	32·4	357	40·9	2957	39·8	3314
Female	66·3	730	58·7	4239	59·7	4969
Age						
M	30·22		29·85		29·91	
sd	5·47		4·79		4·89	
Race/ethnicity						
Asian	4·2	46	3·3	241	3·4	287
Black	33·1	364	23·2	1677	24·5	2041
Hispanic	15·6	172	13·7	992	14·0	1164
White	26·8	295	42·5	3074	40·5	3369
Multi-racial	8·9	98	8·1	582	8·2	680
Other	11·1	122	8·0	575	8·4	697
Married	68·8	757	75·3	5437	74·4	6194
Number of dependent children						
M	1·97		1·74		1·77	
sd	0·99		0·85		0·88	
Dual-earner family	52·6	579	64·6	4668	63·0	5247
Military branch						
Army	50·0	551	27·5	1994	30·6	2545
Air Force	50·0	550	72·4	5231	69·4	5781
Type of service						
Active duty	80·6	888	85·7	6194	85·1	7082
Activated national guard	15·1	166	11·5	830	12·0	996
Activated reserve	3·3	47	2·7	201	3·0	248
Rank						
E-1	0·2	2	0·2	13	0·2	15
E-2	1·4	15	0·4	27	0·5	42
E-3	7·2	79	5·5	398	5·7	477
E-4	27·3	301	19·2	1386	20·3	1687
E-5	35·7	393	35·8	2587	35·8	2980
E-6	28·2	311	38·9	2814	37·5	3125
Reside on an installation	31·5	347	26·6	1922	27·3	2269
Currently experiencing deployment/TDY	11·3	124	11·5	831	11·5	955

TDY, temporary duty assignment.

Some categories do not total to the sample total of 8326 due to missing values.

The ANOVA results demonstrated a statistically significant difference in average financial well-being between military families who used a food distribution resource and those who did not (*F* (1, 8513) = 648·334·47, *P* < 0·001). Those who used a charitable food distribution resource had generally lower financial well-being scores (m = 44·17, sd = 8·93) than those who did not visit a charitable food distribution site (m = 51·07, sd = 8·61).

Complete results for the binomial logistic regression examining military and non-military characteristics as predictors explaining the likelihood of military families’ use of a food distribution resource are shown in Table 2. The Hosmer and Lemeshow test for model fit was not statistically significant (χ^2^(8) = 4·68, *P* = 0·79), indicating that the data were a sufficient fit to the tested model.

**Table 2:** Multiple binary logistic regression assessing demographic and military characteristics associated with utilising charitable food resources

	B	se	OR	95 % CI
Military characteristics				
Rank	−0·37	0·04***	0·69	0·64, 0·75
Currently experiencing military assignment (deployment or TDY)	−0·16	0·11	0·85	0·69, 1·07
Resides on an installation	0·10	0·08	1·11	0·96, 1·29
Army (ref. Air Force)	0·84	0·07***	2·31	2·01, 2·67
Non-military characteristics				
Age	0·03	0·01***	1·04	1·02, 1·05
Race/ethnicity (ref. White)				
Asian	0·53	0·18**	1·69	1·19, 2·40
Black	0·41	0·09***	1·50	1·26, 1·79
Hispanic	0·18	0·12	1·20	0·95, 1·50
Multi-racial	0·38	0·13**	1·47	1·14, 1·89
Other	0·41	0·13**	1·51	1·17, 1·96
Single parent household	0·14	0·10	1·15	0·94, 1·41
Number of dependent children	0·30	0·04***	1·35	1·25, 1·47
Dual-earner family	−0·31	0·10**	0·73	0·61, 0·89

B, beta; se, standard error; OR, odds ratio; TDY, temporary duty assignment.

** *P* < 0·01.

*** *P* < 0·001.

Of the military characteristics examined, for every one-unit increase in service member rank, the odds of using a food distribution resource use were 0·69 times that of lower ranking service members (OR = 0·69, 95 % CI = 0·64, 0·75, *P* < 0·001). This represents a 31 % decrease (1–0·69 = 0·31) in the likelihood of using a food distribution resource for every one-unit increase in rank. Additionally, Army families were 131 % more likely to utilise food distribution resources than Air Force families (OR = 2·31, 95 % CI = 2·01, 2·67, *P* < 0·001). Experiencing a current deployment/TDY and living on a military installation were not associated with food distribution resource use.

After accounting for these military characteristics, older age and having more dependent children were associated with an increased likelihood of food distribution resource use (OR = 1·04, 95 % CI = 1·02, 1·05, *P* < 0·001 and OR = 1·35, 95 % CI = 1·25, 1·47, *P* < 0·001, respectively). More specifically, across the sample, for every one-year increase in age, families’ odds of using food distribution resources were 4 % greater. For every additional dependent child reported, the odds of using food distribution resources were 35 % greater. The odds of utilising food distribution resources were higher for respondents who were Asian, Black, Multi-racial or an ‘other’ racial/ethnic minority (Asian: OR = 1·69, 95 % CI = 1·19, 2·40, *P* = 0·003; Black: OR = 1·50, 95 % CI = 1·26, 1·79, *P* < 0·001; multi-racial: OR = 1·47, 95 % CI = 1·14, 1·89, *P* = 0·003; other: OR = 1·51, 95 % CI = 1·17, 1·96, *P* = 0·002). More specifically, relative to White respondents, Asian respondents had 69 % greater odds of using food distribution resources, followed by 50 % greater odds for Black respondents, 47 % greater odds for multi-racial respondents and 51 % greater odds for ‘other’ racial/ethnic minorities. Although food distribution resource utilisation did not differ between single-parent and married households, two-income families (i.e. dual-earner households) had a 27 % reduction in the odds of utilising these resources compared with single-earner households (OR = 0·73, 95 % CI = 0·61, 0·89, *P* < 0·001).

## Discussion

### Examining the prevalence of food distribution resources and variation by financial well-being

The current study extended previous studies primarily focusing on food insecurity to examine an important related construct, the use of charitable food distribution resources. Furthermore, we focused specifically on military families with young children given that they are a population that is vulnerable to food insecurity^(8)^. Approximately one in eight families (13·2 %) in the current sample reported using food distribution resources to make ends meet within the previous year. To our knowledge, previous research on the prevalence of food distribution resource use among military families with small children is not available as a comparison for these numbers. However, this percentage is notably smaller than the 33 % of military families found to be experiencing marginal food insecurity in a 2022 study^(9)^. Although the 2022 study on food insecurity utilised a different sample, and, therefore, direct comparisons cannot be made, together these results may suggest that only a subset (possibly less than half) of military families who experience food insecurity use food distribution resources. Although this is not necessarily a surprising finding and is consistent with other research noting the ‘gap’ between food insecurity and resource engagement^(21)^, the results provide insight on how military families’ needs align (or do not align) with resource use. Information on the alignment between needs and resource use is an important baseline for maximising the use of food insecurity resources among those who need them.

The current study’s examination of the association between military families’ use of food distribution resources and their financial well-being provides additional insight on how needs align with resource use. As expected, families with lower financial well-being (i.e. demonstrating need) were more likely to use food distribution resources. More specifically, the average CFPB financial well-being score for participating families who did not utilise food distribution resources was 51·07, which is considered ‘medium-high financial well-being’ based on the measure scoring criteria^(22)^. In contrast, those who utilised food distribution resources had an average financial well-being score of 44·17, which is considered ‘medium-low financial well-being.’

### Characteristics associated with variation in resource utilisation

Army families and families with a lower ranking service member were more likely to report utilising food distribution resources, and these differences were quite substantial. Army families were 131 % more likely to utilise food distribution resources compared with Air Force families, and each one-unit increase in rank was related to a 31 % decrease in the likelihood of using food distribution resources. Because the logistic regression model considered the military and non-military characteristics simultaneously, the results identify unique associations between each predictor and food distribution resource use after accounting (i.e. controlling) for the other predictor variables. Consequently, the findings indicate that Army families were more likely to utilise food distribution resources even after accounting for some variables that differ by branch and may be related to resource use (e.g. number of children, single-parent households, dual-earner households and racial/ethnic minorities).

Rank represents the ‘social address’ of military families and is closely tied to service members’ pay as well as social hierarchy^(23)^. Consequently, higher rates of food distribution resource use by families with a lower-ranking service member likely reflect a combination of these factors, including, but not limited to, the service member’s take-home pay amount and perceived social implications connected to resource utilisation. That is, higher ranking service members are less likely to need the resources because of their higher income. At the same time, higher ranking service members may be more hesitant to utilise resources they need due to perceived stigma or career concerns linked to utilising resources^(24)^.

Interestingly, although some research suggests that living on a military installation can increase access to the many resources available to military families (ranging from access to gyms, childcare and family programming)^(25)^, we found the use of food distribution resources did not vary depending on whether families lived on or off the installation. Furthermore, currently experiencing a deployment or TDY was unrelated to food distribution resource use, which aligns with previous research^(1)^. It was suggested that the additional financial compensation typically connected to assignments requiring the service member to be away from their duty station may offset other challenges that increase financial strains during deployment (e.g. childcare needs).

Regarding non-military characteristics examined, older respondents and those with more dependent children were more likely to utilise food distribution resources. Age and the number of dependent children may be conflated, as older service members are more likely to have multiple children. However, the finding that older respondents were more likely to utilise food distribution resources is also consistent with research with other vulnerable groups (e.g. immigrants), where older respondents were more likely to utilise food pantries than younger respondents^(26)^.

Resource utilisation did not vary between single- and multiple-parent households. However, dual-earner families were, on average, 27 % less likely to utilise these resources compared with single-earner families. Given the known challenges that military families face with spousal employment, including higher rates of unemployment and underemployment^(15)^, these findings further support the need for adequate food distribution resources to military families coupled with ongoing efforts to reduce spousal un/underemployment. Members of most racial/ethnic minority groups were approximately 50 % more likely to utilise food distribution resources compared with Whites. An exception was Hispanics, who reported similar utilisation rates as Whites. These findings may reflect larger systematic/cultural differences ranging from discrimination in spousal employment opportunities to cultural expectations to support extended kin in times of need^(27,28)^ that warrant future exploration. The military is an ideal setting for such research, given that there is considerably less variation in the pay structure within the military than in other work settings.

### Strengths and limitations

The current study has numerous strengths, including the large, national sample of military families with young children, which increases the likelihood that these findings are not specific to a certain physical location. The inclusion of both Army and Air Force military families means the findings are not specific to a single branch (though expansion to include other branches would be important in future research). The timing of the data collection is also a strength, given that it occurred approximately one year into the COVID-19 pandemic. Consequently, the study extends previous research before COVID-19 (as well as the studies that occurred in the very early months of the pandemic) to provide a more current ‘snapshot’ of how military families were faring financially.

Of course, there are also study limitations that warrant consideration. Given that the sample was comprised of service members with a rank of E-1 to E-6 (enlisted), it is unclear if these findings apply to officers or warrant officers. The study also utilises data from families who were applying for supplemental childcare funding; thus, it may represent a sample in greater need and/or a sample of families more likely to be aware of community support. Most likely, we would anticipate that the families captured in this study may be more likely than others (e.g. single service members, those with older children) to utilise resources. However, this difference does not appear to be born out in the results. Instead, the prevalence of resource utilisation was relatively low (13·2 %).

### Implications

Food insecurity is a known public health concern and, in connection to service members, it can constitute a national defense concern due to its relation to retention intentions^(10)^. Thus, a better understanding of factors connected to military families’ food insecurity is vital, including understanding the use of resources intended to address food insecurity. Such knowledge has numerous implications for prevention and intervention efforts. Broadly, the relatively small number of families in the current study who utilised such resources may indicate the need to grow resource utilisation. It may be that those who need the resources are not using them. Such efforts could take the form of targeted information campaigns to ensure that those in need (e.g. single-earner families) are aware of the resources and also find ways to offset stigma or other social concerns that may keep those in need from utilising available resources. Collaboration between community resources is also imperative. Formal and informal partnerships between service agencies can help expand knowledge of available supports and encourage the use of diverse resources to support family stability and well-being^(23)^. For example, childcare centres may maintain a list of available resources in their community, including but not limited, to food distribution resources. Centres can educate the families they serve about these sources, and vice versa, food access resource sites can cross-promote other community supports (e.g. the Supplemental Nutrition Assistance Program, access to childcare, respite care).

Further, the findings highlight certain groups who are more likely to utilise resources, which may signal their greater need for additional supports and related interventions (e.g. single-earner families, lower ranks and certain racial/ethnic minorities). The findings highlight the need to continue tackling ‘upstream’ determinants (e.g. economic opportunities) with a multitude of ‘downstream’ effects (e.g. food insecurity, living conditions, but also readiness and resilience for military families)^(24)^. For instance, efforts to address spousal employment concerns can improve military families’ financial well-being and reduce food insecurity (and, consequently, the use of food distribution resources).

### Conclusion

Together, these findings are informative for better understanding of food distribution resource utilisation among military families with young children. They underscore the need of such resources, given the association with financial well-being. The findings also highlight characteristics related to resource distribution. This information can be utilised in prevention and intervention efforts to reduce the need for such resources while simultaneously better connecting those in need to the available resources. Further, the findings provide clear next steps for research identifying how resource utilisation maps onto barriers that hinder resource use and factors that aid in promoting the use of available resources.
